# Effects of Beetroot Juice on Physical Performance in Professional Athletes and Healthy Individuals: An Umbrella Review

**DOI:** 10.3390/nu17121958

**Published:** 2025-06-09

**Authors:** Chen Tian, Qingrui Jiang, Mengke Han, Lu Guo, Ruixin Huang, Li Zhao, Shanshan Mao

**Affiliations:** 1 Beijing Key Laboratory of Sports Performance and Skill Assessment, Beijing Sport University, Beijing 100084, China; 2024210410@bsu.edu.cn (C.T.); zhaolispring@126.com (L.Z.); 2 School of Sports Medicine and Rehabilitation, Beijing Sport University, Beijing 100084, China; qingruijiang@bsu.edu.cn (Q.J.); keera0219@163.com (M.H.); xinrhuang@139.com (R.H.); 3 School of Psychology, Beijing Sport University, Beijing 100084, China; guol@bsu.edu.cn

**Keywords:** beetroot juice, nitrate, athletes, non-athletes, physical performance, umbrella review

## Abstract

**Background:** Beetroot juice, the most commonly used route of dietary nitrate supplementation, is theorized to enhance physical performance. However, its effects on different aspects of physical performance, different populations, and optimal supplementation strategies remain controversial. The purpose of this study was to investigate the effects of beetroot juice (nitrate-rich) on physical performance, to compare its effects between professional athletes and non-athletes (healthy individuals), and to determine the optimal supplementation strategy. **Methods:** A systematic search was conducted on the Web of Science, Embase, PubMed, Cochrane Database, SPORTDiscus, Scopus, and CINAHL databases. Reviewers conducted study screening and selection, data extraction, and assessment of methodological quality using the AMSTAR 2 tool. Fifteen meta-analyses were included in this Umbrella Review. A narrative and quantitative synthesis was performed. Standardized mean differences (SMD) and 95% confidence intervals (CI) were reported. **Results:** (1) Beetroot juice significantly improved muscle strength (SMD = 0.08, *p* < 0.001), but the effect size was negligible. (2) Aerobic Endurance: Beetroot juice significantly improved VO2max (SMD = 0.16, *p* = 0.033) in healthy adults; however, the effect size was negligible. (3) Lactate Tolerance: beetroot juice significantly improved TTE (SMD = 0.25, *p* = 0.034) and YO-YOIR1 (SMD = 0.27, *p* = 0.049) performance in healthy adults, but the effect size was small. (4) Subgroup analyses revealed significant population differences: professional athletes showed significant muscular strength benefits (SMD = 0.27, *p* = 0.007), whereas non-athletes had more pronounced aerobic endurance improvements (SMD = 0.26, *p* < 0.001), but the effect size was small. (5) Nitrate supplementation timing and dose–response analysis revealed that nitrate supplementation, whether administered 2–3 h before exercise or over a prolonged period (≥3 days), produces a significant enhancement in physical performance (*p* < 0.01). Notably, acute improvement only has a small effect size (SMD = 0.20), while the impact of chronic supplementation is essentially negligible (SMD = 0.13). A dosage of 8.3–16.4 mmol NO_3_^−^ (515–1017 mg) showed a significant improvement (SMD = 0.14, *p* = 0.029), although the effect size was negligible. **Conclusions:** Acute (2–3 h pre-exercise) and chronic (≥3 days) supplementation with beetroot juice to achieve nitrate levels of 8.3–16.4 mmol (515–1017 mg/d) are recommended to enhance physical performance. Beetroot juice shows population-specific effects: proper beetroot juice supplementation improves muscular strength in professional athletes and aerobic endurance in non-athletes. Moreover, appropriate supplementation of beetroot juice can improve the lactate tolerance in healthy adults.

## 1. Introduction

In recent years, dietary nitrate has garnered significant attention in exercise science, fitness, and sports nutrition due to its potential ergogenic effects [1]. In high-level competition, even subtle supplement-induced changes can affect the outcome of a competition, even though they can only have a subtle effect on physical performance [2].

Currently, the natural dietary route (especially beetroot juice) is the generally accepted form of nitrate supplementation. Nitrate supplementation through the dietary route is unanimously recommended by experts because of its effectiveness and safety compared to nitrate supplementation alone [3]. Nitrates are bioactive compounds that are widely found in green leafy vegetables and root vegetables [4]. Of the many sources of dietary nitrate, beetroot juice is the most widely studied form of supplementation [5,6]. The reasons are as follows: 1. Beetroot juice contains a high concentration (250–500 mg/100 g) of nitrate (NO_3_^−^) [7]. 2. Nitrate bioavailability of beetroot is significantly better than that of other vegetables [8,9,10]. Moreover, some studies comparing regular beetroot juice with nitrate-removed beetroot juice have shown that the regular beetroot juice group significantly improved physical performance [11,12]. Additionally, studies have shown that compared to an equivalent dose of sodium nitrate (both containing 6 mmol of nitrate), nitrate-rich beetroot juice can reduce oxygen consumption during exercise by 4% [13], which may be attributed to the role of antioxidants in vegetables in promoting NO production [14].

The biological activity of dietary nitrates (such as those present in beetroot juice) is predominantly mediated by their metabolic conversion to nitric oxide (NO) in vivo. Nitric oxide (NO) is an important signaling molecule and regulatory factor, with its vasodilatory properties being well-documented in numerous studies [15,16]. The synthesis of NO primarily occurs through two pathways: the first involves an oxygen-dependent reaction catalyzed by the nitric oxide synthase (NOS) family, which converts L-arginine into NO [17]; the second is the nitrate–nitrite–NO pathway, in which dietary nitrate serves as a precursor to NO. Upon ingestion, nitrate is extracted and concentrated in saliva, where it is subsequently reduced to nitrite by anaerobic bacteria in the oral cavity. After absorption in the gastrointestinal tract, nitrite is further reduced to NO [14,18,19]. In recent years, an increasing body of research has demonstrated the therapeutic potential and ergogenic effects of NO in both cardiovascular and metabolic diseases [20], as well as in physical performance [10]. Specifically, NO enhances physical performance through mechanisms such as regulating muscle function, influencing glucose uptake and metabolic processes, facilitating muscle damage repair, and modulating inflammatory responses [21].

However, the efficacy of beetroot juice supplementation for improving physical performance in athletes and non-athletes remains controversial. Some studies have indicated that dietary nitrate supplementation, primarily in the form of beetroot juice, effectively enhances physical performance, particularly aerobic endurance and exercise economy [12,22,23,24]. However, some studies have reported no significant improvements in aerobic endurance performance following dietary nitrate supplementation [25,26,27,28,29]. While early research primarily focused on the effects of dietary nitrate on aerobic endurance and exercise economy, recent studies have increasingly examined its influence on other performance aspects, such as muscle strength [30,31,32,33], balance ability [34], neuromuscular function [35,36], and fatigue recovery [24], all of which can impact physical performance and outcomes. Nevertheless, findings regarding the effectiveness of beetroot juice (BRJ) in these areas remain inconclusive. Some studies have reported that beetroot supplementation (BRS) improved muscle endurance, manifesting as an increase in repetitions or prolonged time to fatigue [24,31,33,37,38,39,40]. Conversely, other studies have found no such effects [32,41,42,43,44]. Regarding muscle strength—defined as the maximum force produced by a muscle during a single maximal effort—BRS appears to have no significant impact on maximum strength performance under resting conditions [32,41,44], but it may attenuate strength loss following exhaustive exercise [24,45,46,47]. Notably, the variability in the ergogenic effects of dietary nitrate on physical performance may be influenced by multiple factors, including training status, type of exercise, muscle groups involved, nitrate dosage, experimental conditions, and individual variability [30].

The effects of dietary nitrate supplementation differ significantly between athletes and non-athletes. Studies focusing on elite athletes have indicated that dietary nitrate supplementation does not significantly enhance performance or competitive outcomes [35,48,49,50,51,52]. These studies encompass various sports, including rugby [51], triathlon [50], field hockey [35], and swimming [52]. Additionally, systematic reviews have drawn similar conclusions. For instance, the study by Campos et al. [53] suggested that non-athletes may benefit more from nitrate (NO_3_^−^) supplementation, whereas elite athletes, due to their already high fitness levels, derive fewer performance benefits. Other studies have shown that dietary nitrate supplementation enhances performance in individuals with low to moderate training levels [54] but does not yield significant improvements in highly trained endurance athletes [10]. However, some research has shown that dietary nitrate supplementation can improve elite athletes’ performance [55,56,57]. Therefore, one of the primary objectives of this study was to explore the differential effects of beetroot juice supplementation on physical performance between athletes and non-athletes.

Supplementation strategies play a crucial role in determining the efficacy of dietary nitrate on physical performance, with factors such as dosage and timing being particularly influential. Although previous studies have examined various supplementation strategies and proposed optimal protocols [8,58,59], significant discrepancies exist across studies, potentially due to differences in experimental design, participant characteristics, or exercise modalities. For example, the study by Wylie et al. [8] investigated the effects of three different doses (4.2 mmol, 8.4 mmol, and 16.8 mmol NO_3_^−^) of NO_3_^−^ on physical performance. Their findings revealed that low-dose (4.2 mmol NO_3_^−^) supplementation consumed 2.5 h before a TTE (time to exhaustion) test did not significantly improve performance, whereas moderate (8.4 mmol NO_3_^−^) and high (16.8 mmol NO_3_^−^) doses increased TTE by 14% and 12%, respectively. However, a meta-analysis suggested that an acute nitrate dosage of 5–14.9 mmol, administered at least 150 min before exercise, may be the most effective strategy [60]. Furthermore, this study found that when supplementation exceeds two days, the relationship between dosage and performance enhancement becomes negatively correlated, with an optimal dosage range of 5–9.9 mmol/d. These inconsistent findings highlight the need for further research to elucidate the mechanisms underlying the effects of different supplementation strategies on physical performance, ultimately optimizing dietary nitrate supplementation protocols.

Due to the discrepancies in the research results regarding the enhancement of physical performance by beetroot juice, including different aspects of performance (strength, speed, and cardiorespiratory endurance), diverse supplementation schemes (dosage and timing), and various populations (athletes and non-athletes), the purpose of this umbrella review is to synthesize existing evidence, explore different aspects of physical performance and population-specific differences in the effects of beetroot juice, and to determine the optimal supplementation strategy.

## 2. Materials and Methods

This umbrella review was conducted and reported in accordance with the guidelines outlined in the PRISMA (Preferred Reporting Items for Systematic Reviews and Meta-Analyses) statement [61]. The study protocol was registered in PROSPERO (Registration Number: CRD42025636403).

### 2.1. Inclusion and Exclusion Criteria

The selection and exclusion criteria for all relevant articles were determined by two researchers (C.T. and Q.R.J.).

Inclusion Criteria: We included peer-reviewed, full-text meta analyses with quantitative synthesis (≥2 studies) published in English; (1) healthy adults or trained athletes aged 18–65 years with no known diagnosed disorders; (2) assessed dietary-nitrate supplementation delivered specifically as beetroot juice or an equivalent nitrate-rich formulation, administered either acutely (a single dose 2–3 h before exercise) or chronically (for ≥3 days) at a dose of ≥4.6 mmol nitrate per day or per administration; (3) compared the intervention with a placebo, a low-nitrate control, or no-supplement condition; and (4) reported at least one validated physical-performance endpoint, namely muscular strength or endurance, maximal oxygen uptake (VO_2_ max), lactate-tolerance measures such as time to exhaustion (TTE) or the Yo-Yo intermittent-recovery test level 1, or speed assessed by a time-trial. Only the most complete dataset was retained when multiple publications described the same cohort. Exclusion Criteria: We excluded (1) duplicate publications, Scoping reviews, narrative reviews, protocols, conference abstracts, primary RCTs, reviews without a meta-analysis; (2) articles whose full text was inaccessible; (3) trials that tested multi-ingredient formulas without a dedicated nitrate arm or employed active comparators lacking a true placebo/low-nitrate control; (4) children, elderly with chronic diseases, or any participants with diagnosed health conditions; studies mixing healthy and clinical populations without separable data; and (5) reports that failed to provide or allow extraction of the specified performance outcomes, or reported only biochemical/surrogate markers. Information about inclusion and exclusion criteria can be found in Appendix A.

### 2.2. Search Strategy

A comprehensive search was conducted on the Web of Science, Embase, PubMed, Cochrane Database, SPORTDiscus, Scopus, and CINAHL databases, covering the period from 2000 to November 2024. A search strategy was employed, utilizing a systematic review filter to refine the search results. Regular updates to the search were performed to identify the most recently published systematic reviews. The search was conducted using subject headings and free-text terms related to beetroot, nitrate, and exercise. A detailed search strategy is provided in Supplementary File S1. The search strategy was adapted for different databases to ensure comprehensive coverage.

### 2.3. Study Selection

Following the completion of the literature search, reference management software (EndNote 20) was used to organize the studies. After removing duplicate records, two reviewers (C.T. and Q.R.J.) screened the titles and abstracts to exclude ineligible studies. Full-text screening of the eligible studies was then conducted by the same two independent reviewers. Any discrepancies were resolved by consulting a senior third reviewer (S.M.).

### 2.4. Data Extraction

Data were extracted for the following items: authors of the review; publication date of the review; number of included studies; characteristics of the included population in the review; intervention methods of the studies; timing and dosage of dietary nitrate supplementation; and results of the review. The data extraction form used is presented in Table 1.

### 2.5. Quality Assessment

The methodological quality of the included systematic reviews was assessed using the A Measurement Tool to Assess systematic Reviews 2 (AMSTAR 2) checklist [73]. The reviews were classified into four categories: high, moderate, low, and critically low quality. Among the 16 items on the AMSTAR 2 checklist, 7 were identified as critical domains for evaluating methodological rigor. These key domains include the following: registering the review protocol before initiation, conducting a comprehensive literature search, providing justifications for excluding individual studies, adequately assessing the risk of bias in included studies, using appropriate statistical methods in meta-analyses, and considering the risk of bias when interpreting results. If any key domain is not met, the study was rated as low quality; studies rated as very low quality were excluded. Detailed information on the quality assessment is provided in Supplementary File S2, Table 1.

### 2.6. Risk of Bias Assessment

Publication bias occurs when studies with significant results are favored over those with non-significant findings, potentially influencing data analysis. Funnel plots were used to assess this bias by plotting sample size (or inverse standard error) against effect size. Larger studies yield more precise effect estimates, forming a funnel shape. An asymmetric funnel indicates possible publication bias, which was statistically tested using Egger’s test [74]. The detailed risk of bias results are presented in Supplementary File S3.

### 2.7. Reviews with Overlapping Primary Studies

The fundamental characteristics of the included reviews were reported in tabular format, specifying which outcomes were addressed in each review. To assess the degree of overlap among systematic reviews and evaluate potential redundancy, the Corrected Covered Area (CCA) Index was utilized. The extent of literature duplication was classified into four categories: (1) CCA: 0–5% (Minimal Overlap)—indicating a low degree of overlap among systematic reviews, with minimal duplication of primary literature; (2) CCA: 6–10% (Moderate Overlap)—representing a moderate level of overlap that requires researchers’ attention to potential redundancy; (3) CCA: 11–15% (High Overlap)—suggesting a substantial degree of overlap, with multiple systematic reviews sharing a significant portion of the primary literature; and (4) CCA: >15% (Extremely High Overlap)—indicating severe overlap among systematic reviews, with a considerable amount of duplicate primary literature. In cases where overlap falls within this range, researchers should conduct a rigorous assessment before selecting systematic reviews for inclusion [75,76]. The detailed process and results are presented in Supplementary File S4.

### 2.8. Data Synthesis

The data synthesis was descriptive and presented in detailed tabular summaries. The extracted data were consistently analyzed for quantitative synthesis, and standardized mean differences (SMD) with 95% confidence intervals (CI) were calculated. In addition, following Cochrane’s recommended harmonized SMD directions [77]. Data analysis was performed using R (version 4.4.2) and STATA 17 (METAN package), employing both random-effects and fixed-effects models to compute the standardized mean differences. The overall effect size (95% CI) and *I*^2^ statistic [78] were calculated using STATA17 (METAN package). Effect sizes were categorized as negligible (<0.2), small (<0.59), moderate (<1.19), and large (>1.19) [79]. The *I*^2^ values were interpreted as follows: low heterogeneity (*I*^2^ < 25%), moderate heterogeneity (25% ≤ *I*^2^ < 50%), and significant heterogeneity (*I*^2^ ≥ 50%) [80]. The detailed data processing results are presented in Supplementary Files S3 and S5.

## 3. Results

### 3.1. Literature Search

**Figure 1:**
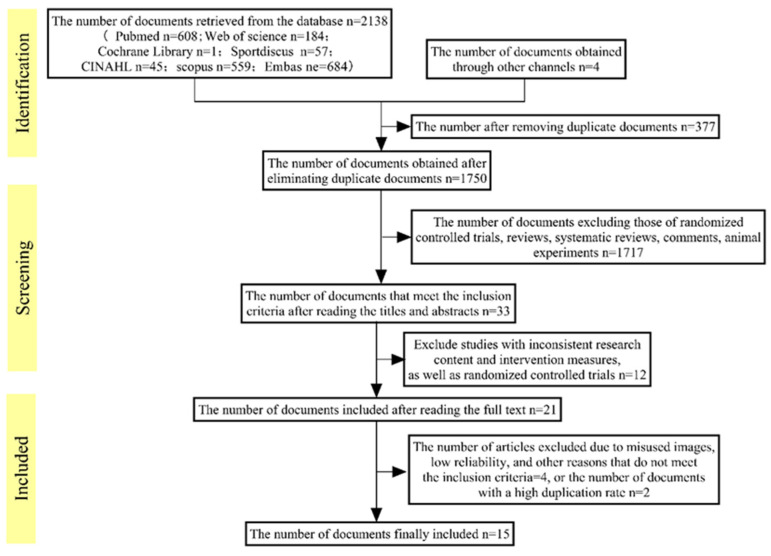
Study selection process following the PRISMA flowchart.

A total of 2138 articles were retrieved from the Web of Science, Embase, PubMed, Cochrane Database, SPORTDiscus, Scopus, and CINAHL databases. After excluding 377 duplicate articles, 21 articles were selected by reviewing the titles and abstracts of the remaining articles. Based on the inclusion and exclusion criteria, 4 articles were excluded after full-text review, 0 articles were excluded due to low quality, and 2 articles were excluded due to their impact on the duplication rate of the original literature. Ultimately, 15 studies were included in this umbrella review. Figure 1 illustrates the selection process according to the PRISMA flowchart.

### 3.2. Quality Assessment

All 15 included systematic reviews met the quality requirements, as assessed using the AMSTAR 2 tool. The majority of the reviews were rated as moderate to high in methodological quality.

### 3.3. Summary of the Results

**Figure 2:**
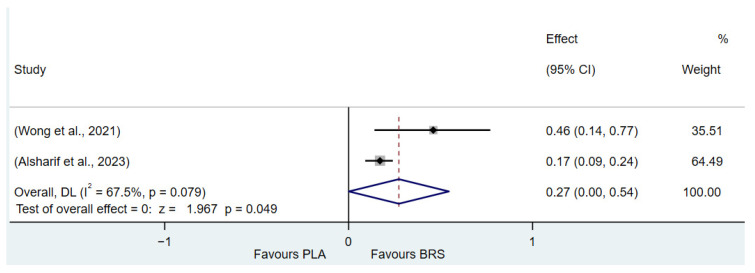
Forest plot of the effects of beetroot juice supplementation on Yo-Yo IR1 [59,69]. The vertical red dashed line marks the overall standardised mean difference (pooled effect size).

**Figure 3:**
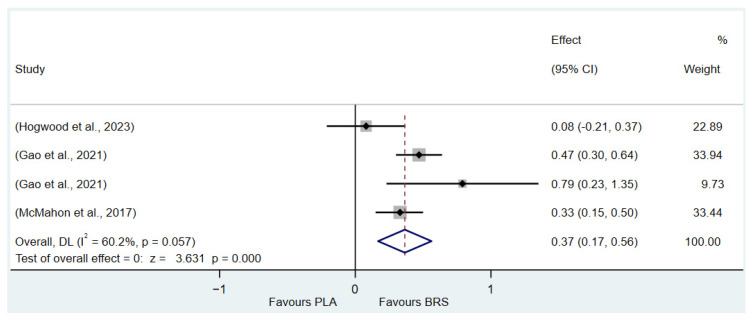
Forest plot of the effects of beetroot juice supplementation on time to exhaustion in seconds [65,70,72]. The vertical red dashed line marks the overall standardised mean difference (pooled effect size).

**Figure 4:**
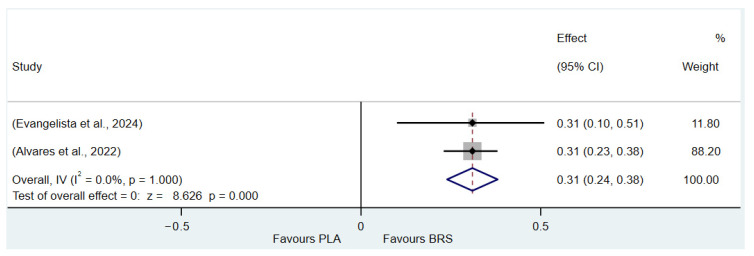
Forest plot of the effects of beetroot juice supplementation on muscle endurance [58,64]. The vertical red dashed line marks the overall standardised mean difference (pooled effect size).

**Figure 5:**
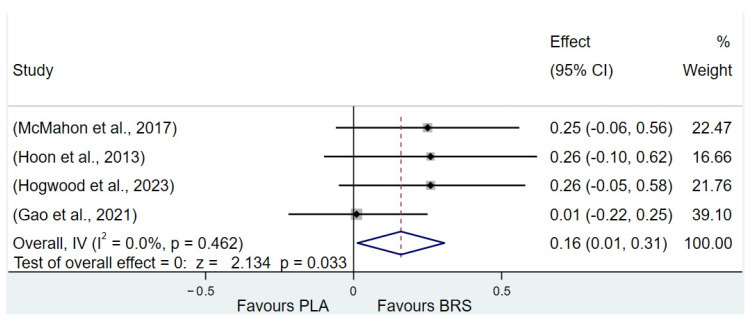
Forest plot of the effects of beetroot juice supplementation on maximal oxygen uptake (VO_2_ max) [65,70,71,72]. The vertical red dashed line marks the overall standardised mean difference (pooled effect size).

**Figure 6:**
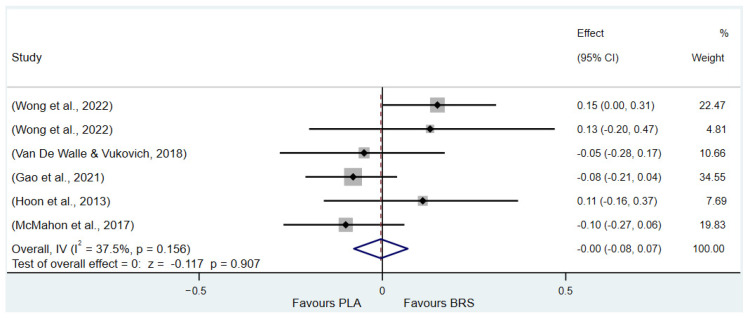
Forest plot of the effects of beetroot juice supplementation on time-trial [62,68,70,71,72]. The vertical red dashed line marks the overall standardised mean difference (pooled effect size).

**Figure 7:**
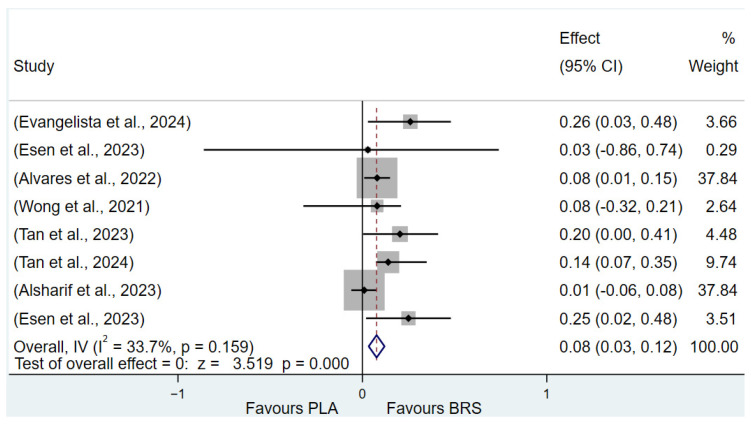
Forest plot of the effects of beetroot juice supplementation on muscle strength [51,58,59,64,66,67,69]. The vertical red dashed line marks the overall standardised mean difference (pooled effect size).

**Figure 8:**
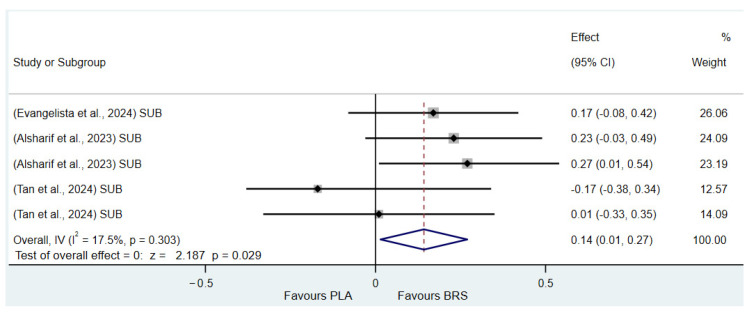
Forest plot showing the effects of 8.3–16.4 mmol/d of NO_3_^−^ on physical performance [58,59,66]. The vertical red dashed line marks the overall standardised mean difference (pooled effect size).

**Figure 9:**
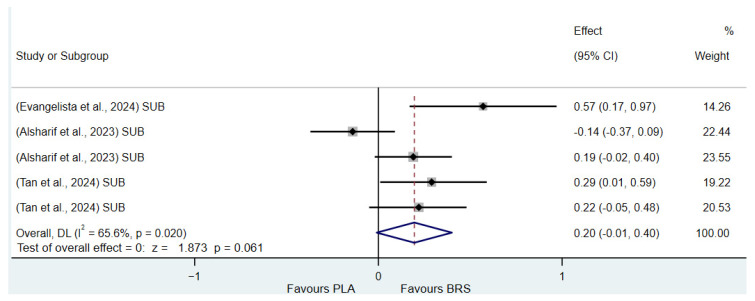
Forest plot showing the effects of 4.6–8.3 mmol/d of NO_3_^−^ on physical performance [58,59,66]. The vertical red dashed line marks the overall standardised mean difference (pooled effect size).

**Figure 10:**
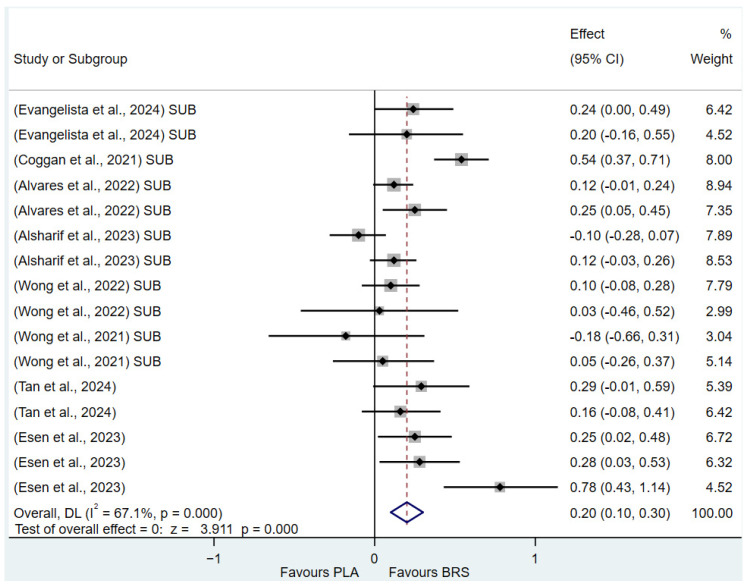
Forest plot of the effects of acute beetroot juice supplementation (2–3 h before exercise) on physical performance [51,58,59,63,64,66,68,69]. The vertical red dashed line marks the overall standardised mean difference (pooled effect size).

**Figure 11:**
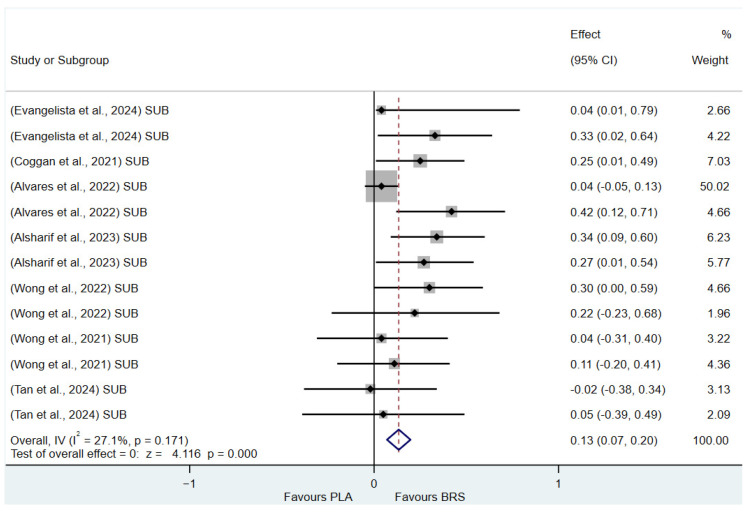
Forest plot of the effects of chronic beetroot juice supplementation (≥3 days) on physical performance [58,59,63,64,66,68,69]. The vertical red dashed line marks the overall standardised mean difference (pooled effect size).

**Figure 12:**
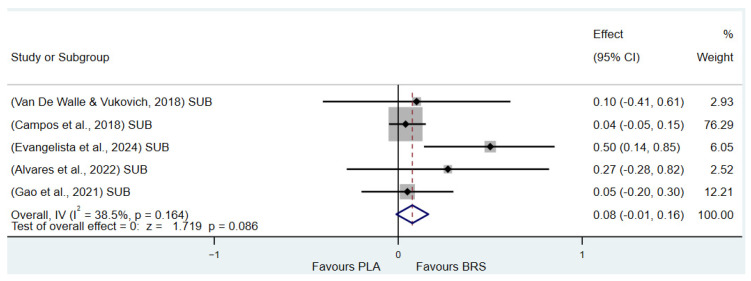
Forest plot of the effects of beetroot juice supplementation on aerobic endurance in professional athletes [53,58,62,64,72]. The vertical red dashed line marks the overall standardised mean difference (pooled effect size).

**Figure 13:**
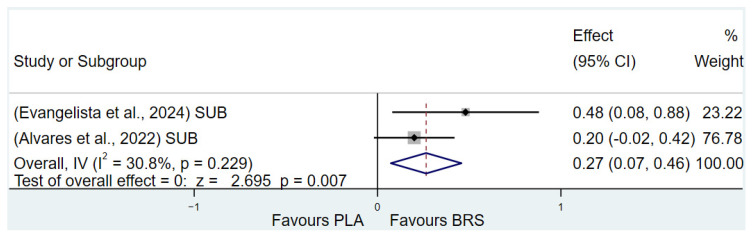
Forest plot of the effects of beetroot juice supplementation on muscle strength in professional athletes [58,64]. The vertical red dashed line marks the overall standardised mean difference (pooled effect size).

**Figure 14:**
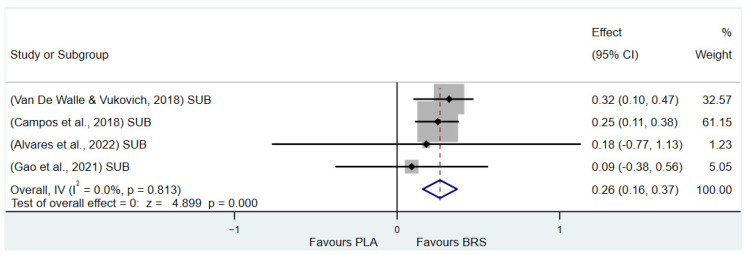
Forest plot of the effects of beetroot juice supplementation on aerobic endurance in non-athletes [53,62,64,72]. The vertical red dashed line marks the overall standardised mean difference (pooled effect size).

**Figure 15:**
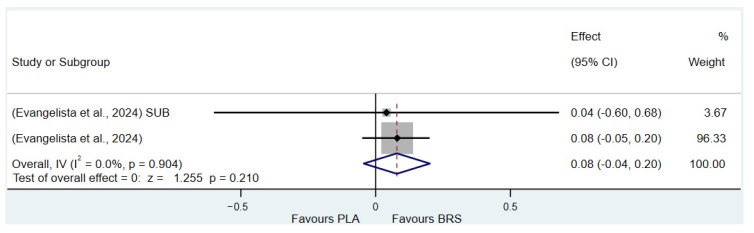
Forest plot of the effects of beetroot juice supplementation on muscle strength in non-athletes [58]. The vertical red dashed line marks the overall standardised mean difference (pooled effect size).

This umbrella review excluded two meta-analyses with a high proportion of duplicate original studies and reanalyzed the remaining studies. The Corrected Covered Area (CCA) Index was determined to be 7.07%, indicating a moderate degree of overlap. Despite this moderate overlap, the complexity of the research field necessitates the inclusion of different meta-analyses, as the original studies included in each meta-analysis vary in terms of outcome measures and study populations. These differences provide a broader perspective for a comprehensive understanding of the research question and contribute valuable information that cannot be simply disregarded by eliminating duplicate studies. While the presence of duplicate studies may introduce certain challenges, such as affecting the accuracy and interpretability of the results and increasing analytical complexity, a comprehensive synthesis of these studies allows for a deeper exploration of the multidimensional aspects of the research question. This approach enables the identification of commonalities and distinctions among different studies, ultimately offering a more comprehensive reference for future research. The details of the outcome indicator data analytics are presented in Figure 2, Figure 3, Figure 4, Figure 5, Figure 6, Figure 7, Figure 8, Figure 9, Figure 10, Figure 11, Figure 12, Figure 13, Figure 14 and Figure 15.

#### 3.3.1. Muscular Fitness (All Healthy Adults)

Muscle fitness primarily consists of two key components: muscular endurance and muscular strength [81]. The results of the study showed that beetroot juice supplementation significantly improved muscular endurance (SMD: 0.31, 95% CI: 0.24 to 0.38, *p* < 0.001, *I*^2^ = 0%), but the effect size was small. This study also found that beetroot juice significantly improved muscle strength (SMD: 0.08, 95% CI: 0.03 to 0.12, *p* < 0.001, *I*^2^ = 33.7%), but the effect size was negligible.

Detailed information on the results of muscle fitness is presented in Figure 4 (muscular endurance) and Figure 7 (muscular strength).

#### 3.3.2. Aerobic Endurance (All Healthy Adults)

The results of the study showed that beetroot juice supplementation significantly improved maximal oxygen uptake (VO2max) in healthy adults (SMD: 0.16, 95% CI: 0.01 to 0.31, *p* = 0.033, *I*^2^ = 0%), but the effect size was negligible. Detailed information on the results of aerobic endurance is presented in Figure 5 (VO2max).

#### 3.3.3. Lactate Tolerance (All Healthy Adults)

In this study, TTE and Yo-Yo IR1 were used as indicators to evaluate lactate tolerance. The results of the study showed that beetroot juice supplement significantly improved TTE (SMD: 0.37, 95% CI: 0.17 to 0.56, *p* < 0.001, *I*^2^ = 67.5%) and Yo-Yo IR1 (SMD: 0.27, 95% CI: 0.00 to 0.54, *p* = 0.049, *I*^2^ = 67.5%) performance, but the effect size was small.

Detailed information on the results of lactate tolerance is presented in Figure 2 (Yo-Yo IR1) and Figure 3 (TTE).

#### 3.3.4. Speed (All Healthy Adults)

Based on the outcome measures included in the studies, this research used the time trial (TT) as an indicator to evaluate speed performance. There was no statistically significant improvement in TT performance (SMD: 0.02, 95% CI: –0.04 to 0.09, *p* = 0.480, *I*^2^ = 37.5%).

Detailed information on the speed performance results is presented in Figure 6 (TT).

#### 3.3.5. Subgroup Analysis

(1) Effect on Muscular Strength in Professional Athletes and Non-Athletes: Beetroot juice significantly improved muscular strength in professional athletes (SMD: 0.27, 95%CI: 0.07–0.46, *p* = 0.007, *I*^2^ = 30.8%); however, the effect size was small. In contrast, no statistical improvement was observed in non-athletes (SMD: 0.08, 95% CI: −0.04 to 0.20, *p* = 0.210, *I*^2^ = 0%).
(2) Effect on Aerobic Endurance in Professional Athletes and Non-Athletes: Beetroot juice significantly improved aerobic endurance in non-athletes (SMD: 0.26, 95% CI: 0.16–0.37, *p* < 0.001, *I*^2^ = 0%); yet the effect size was small. In professional athletes, no statistical improvement was observed (SMD: 0.08, 95% CI: −0.01 to 0.16, *p* = 0.086, *I*^2^ = 38.5%).
(3) Effect of Dosage (supplementation of beetroot juice to achieve a certain level of nitrates): A dosage of 4.6–8.3 mmol/d (285–515 mg/d) of NO_3_^−^ exhibited marginal significance [82,83] (SMD: 0.20, 95% CI: −0.01 to 0.40, *p* = 0.061, *I*^2^ = 65.6%), but the effect size was small. A higher dosage of 8.3–16.4 mmol/d (515–1017 mg/d) of NO_3_^−^ demonstrated statistical improvement (SMD: 0.14, 95% CI: 0.02–0.27, *p* = 0.029, *I*^2^ = 17.5%); however, the effect size was negligible.
(4) Acute vs. Chronic Supplementation: Acute beetroot juice supplementation (2–3 h before exercise) showed statistical improvement on physical performance (SMD: 0.20, 95% CI: 0.10–0.30, *p* < 0.001, *I*^2^ = 67.1%), but the effect size was small. Chronic supplementation (continuous intake over multiple days) also showed statistical improvement (SMD: 0.13, 95% CI: 0.07–0.20, *p* < 0.001, *I*^2^ = 27.1%), but the effect size remained negligible.

Detailed information on the results of subgroup analysis is presented in Figure 8 (8.3–16.4 mmol/d of NO_3_^−^), Figure 9 (4.6–8.3 mmol/d of NO_3_^−^), Figure 10 (acute), Figure 11 (chronic), Figure 12 (aerobic endurance in professional athletes), Figure 13 (muscle strength in professional athletes),Figure 14 (aerobic endurance in non-athletes), and Figure 15 (muscle strength in non-athletes).

#### 3.3.6. Heterogeneity

(1) Muscular strength in professional athletes, aerobic endurance in professional athletes, and time trial (TT) showed moderate heterogeneity.
(2) The time to exhaustion test (TTE), Yo-Yo IR1 test, 4.6–8.3 mmol/d dose of NO_3_^−^, and acute beetroot juice supplementation showed significant heterogeneity.

## 4. Discussion

Beetroot juice, as a common dietary nitrate supplement, theoretically has the potential to enhance physical performance. However, findings in the literature remain mixed and controversial. Through a comprehensive analysis of relevant studies, this research evaluated the effects of beetroot juice on overall physical performance. It was found that supplementation 2–3 h before exercise, or chronic supplementation for ≥3 days, with a nitrate content ranging from 8.3 to 16.4 mmol, is required to improve performance. The study also identified significant specificity regarding performance indicators and population differences: professional athletes experienced notable improvements in muscle strength, whereas non-athletes significantly benefited in aerobic endurance. Additionally, beetroot juice improved overall lactate tolerance for both athletes and non-athletes.

### 4.1. Muscle Strength

The results of this study showed that beetroot juice supplementation did not enhance muscle strength for healthy adults (including athletes and non-athletes). Subgroup analysis indicated that this general ineffectiveness resulted primarily from a lack of effects for non-athletes, while supplementation did improve muscle strength for professional athletes.

After beetroot juice supplementation, dietary nitrate is converted into NO in the body [18], potentially enhancing muscle strength via the following mechanisms: 1. Enhanced calcium release: NO may directly promote calcium release from the sarcoplasmic reticulum through transnitrosylation of ryanodine receptors [84], thereby improving muscle strength. 2. Increased contractile force of type II (fast-twitch) muscle fibers: Type II fibers typically exhibit lower microvascular oxygen tension during contraction [85,86]. Under these hypoxic conditions, nitrite can be reduced to NO via deoxyhemoglobin or xanthine oxidoreductase [19,87]. Additionally, NO may upregulate the expression of calcium-handling proteins in type II fibers [85,88]. 3. Vasodilation and improved muscle oxygenation: NO also induces vasodilation, enhancing muscle blood flow and oxygen delivery [15,16].

Possible explanations include significant population specificity in the effects of beetroot juice on muscle strength: 1. In daily physical activities (such as cycling, jogging, and brisk walking), ordinary individuals primarily engage in moderate- to low-intensity aerobic exercise [89], which relies on the aerobic metabolic system. In such activities, energy supply is sufficient, and oxygen demand and supply remain relatively balanced [90]. Conversely, elite athletes (such as weightlifters and sprinters) focus more on resistance training, which predominantly relies on anaerobic metabolism [91,92]. This type of high-intensity exercise is more likely to induce local muscle hypoxia due to inadequate oxygen supply [93], accompanied by lactate accumulation and metabolic stress [94]. In such training scenarios, beetroot juice, rich in nitrates, can activate the nitric oxide (NO) pathway, leading to vasodilation that improves local blood flow [95]. Consequently, this helps to precisely alleviate hypoxia, accelerate the clearance of metabolic waste, and ultimately enhance muscle fatigue resistance [96,97]. 2. The exertion of muscle strength largely depends on rapid energy supply and neuromuscular coordination [98]. Professional athletes may gain more from the conversion of beetroot juice to NO. In contrast, the neuromuscular system of non-athletes may not yet require such optimization mechanisms, or their exercise intensity may not be sufficient to induce significant changes.

Although the present study did not observe improved muscle strength for non-athletes following beetroot juice supplementation (consistent with several previous studies [32,37,44]), other studies have reported contrasting results [31,99]. These discrepancies may stem from differences in muscle strength testing protocols (e.g., faster contraction speeds potentially activating more type II muscle fibers) and variations in participant characteristics. Therefore, further research addressing these factors is warranted.

### 4.2. Aerobic Endurance

This study employed maximal oxygen uptake (VO2max) as the marker of aerobic endurance. The results indicated that beetroot juice supplementation produced a statistically significant improvement (*p* = 0.033) in VO2max among healthy adults; however, the effect size remained below a meaningful threshold (SMD < 0.2). However, subgroup analysis revealed significant population differences: this ineffectiveness was attributed to the inclusion of professional athletes. This study included a total of four studies related to maximal oxygen uptake, two of which reported GXT [70,71] performance (the common method for determining VO2max in a laboratory setting), one directly reported maximal oxygen uptake [72], and another reported peak oxygen uptake [65] (some subjects were elderly, and peak oxygen uptake can be used to assess the level of cardiorespiratory endurance in this population [100]).

Consistent with the results of this study, several studies have confirmed that beetroot juice supplementation can improve aerobic endurance in healthy adult populations [9,12]. The following mechanisms may account for these observed effects: 1. Beetroot juice is reduced to nitrite (NO_2_^−^) by oral bacteria and then converted to NO [95] in the body. NO, as a potent vasodilator, acts on vascular smooth muscle to cause dilation, thereby reducing peripheral resistance and increasing blood flow to skeletal and cardiac muscles [14,16,21]. 2. NO can regulate mitochondrial respiratory efficiency [19]. It reduces proton (H⁺) leak across the mitochondrial inner membrane, thereby enhancing oxidative phosphorylation efficiency [101]. 3. Under hypoxic conditions induced by intense exercise, such as within the microenvironment of type II muscle fibers, nitrite (NO_2_^−^) can act as an alternative electron acceptor in the mitochondrial respiratory chain, theoretically substituting for oxygen [102].

Beetroot juice was effective for improving aerobic endurance for non-athletes but not for athletes, consistent with findings from previous studies [14,53,60,62,87]. The reasons for this discrepancy may be as follows: 1. Significant population differences in aerobic endurance were also observed in this study, a possible explanation for this discrepancy is that individuals with higher aerobic capacity tend to exhibit in-creased expression of nitric oxide synthase (NOS) enzymes [103], thereby enhancing their ability to produce NO via the L-arginine pathway. Additionally, professional athletes typically undergo training adaptations that enhance capillary function, allowing them to maintain muscle oxygenation and upregulate NOS activity under most conditions. Consequently, they exhibit a reduced dependence on the nitrate–nitrite–NO pathway [104]. This physiological adaptation results in higher baseline plasma nitrite levels in professional endurance athletes compared to untrained individuals [103]. Supporting this, Poveda et al. [105] found that endurance-trained athletes had 158% higher plasma nitrite concentrations than their untrained counterparts. As a result, nitrate supplementation does not further increase NO production in these individuals. 2. Genetic and training adaptations influencing VO_2_ max: VO_2_ max is significantly influenced by genetic factors [106]. Athletes typically possess an inherently higher maximal oxygen uptake compared to non-athletes, and after long-term intensive training, their VO_2_ max may approach an individual’s physiological upper limit [107]. Thus, performance-enhancing supplements such as beetroot juice may have minimal additional impact on their maximal oxygen uptake.

### 4.3. Lactate Tolerance

This study evaluated lactate tolerance using the TTE and Yo-Yo IR1 tests. Results indicated that beetroot juice supplementation significantly improved performance in both TTE and Yo-Yo IR1 tests. The results of some studies are consistent with the present study [70,71,72]. This may be because after supplementation with beetroot juice, NO causes dilation of capillaries or small arteries, increases microcirculatory blood flow, improves the transport of tissue oxygen, nutrients, and metabolic wastes [16,21,97,108], accelerates the rate of lactic acid clearance, and allows for a more adequate supply of oxygen to the tissues, as well as mediating an increase in mitochondrial efficiency [19,96,109] (the detailed mechanism is mentioned in the previous section). The above mechanisms result in lower oxygen consumption by the muscles at the same exercise intensity, reduced lactic acid production, increased metabolism, and delayed onset of fatigue.

These findings suggest that beetroot juice supplementation enhances performance not only by optimizing oxygen delivery and utilization efficiency, but also by improving lactate tolerance and delaying the onset of fatigue. These benefits are particularly relevant for high-intensity interval training and competitive sports performance. Although the TTE and Yo-Yo IR1 studies did not differentiate between athletes and non-athletes, athletes likely have a greater demand for such supplements than non-athletes, who prioritize health promotion and exercise experience. Therefore, future research should specifically investigate the effects of beetroot juice supplementation in professional athletic populations.

### 4.4. Speed Performance

In this study, time trials (TTs) were used as a metric to evaluate speed performance.

A time trial (TT) assesses the actual exercise efficiency through exercise performance over a fixed distance or time, and is able to objectively integrate physiological abilities and psychological factors to respond to the subject’s true exercise performance [110]. Although the present study found no significant improvement in TT performance following beetroot juice supplementation, several inherent factors may influence TT results, including the following: 1. participants’ psychological conditions [111], 2. environmental factors [112], and 3. energy reserves and nutritional status during testing [113]. The study also considered its small sample size, variations in testing protocols (e.g., long- vs. short-distance), and differences in subject characteristics (such as age and occupation), since these factors may account for the moderate heterogeneity observed in TT results.

In summary, although TT was used in this study to evaluate speed performance in healthy adults, this metric does not provide a comprehensive measure of speed capability. Further research is necessary to address this limitation in the future.

### 4.5. Beetroot Juice Supplementation Strategies

This study recommends beetroot juice as a route to nitrate supplementation to improve physical performance. As discussed earlier, dietary nitrate supplementation, particularly through beetroot juice, offers greater physiological advantages compared to isolated nitrate supplementation [114]. Numerous studies have shown that nitrate supplementation alone does not enhance physical performance, whereas beetroot juice exhibits ergogenic effects [54,115,116,117,118,119,120]. This discrepancy may stem from the unique phytochemical composition of beetroot juice. It is rich in antioxidants, such as betalains, which not only facilitate the reduction in nitrate to promote NO production but may also enhance performance through multi-target mechanisms, such as modulating oxidative stress and energy metabolism [14,121]. Furthermore, beetroot juice contains about 6–10% carbohydrates, the main component being sucrose (more than 80%) [6], which can serve as an immediate energy substrate during exercise, in addition to providing dietary nitrate. However, as also mentioned in the introduction, some studies have compared nitrate-removed beetroot juice with normal beetroot juice and found that nitrate-removed beetroot juice did not improve physical performance [11,12]. In summary, although it cannot be concluded that beetroot juice improves physical performance solely due to the abundance of nitrates in it, NO_3_^−^ is indispensable for its performance-enhancing effects. At the same time, the synergistic effects of the various compounds in beetroot juice may amplify its performance-enhancing effects [13,122].

Regarding the supplementation dose of beetroot juice, in order to improve physical performance, this study recommends supplementation with beetroot juice resulting in nitrate levels of 8.3–16.4 mmol/d (515–1017 mg/d NO_3_^−^). Current research has not yet reached a consensus on the optimal dose of beetroot juice supplementation. Studies utilizing lower doses (4–5.5 mmol of NO_3_^−^) have reported either no effect on physical performance [8,37,123] or a positive impact [54,124,125,126]. Similarly, studies administering higher doses (>10 mmol) have yielded conflicting results, showing either no effect [41,127] or a beneficial impact on performance [8,128]. In this study, based on the data from the included research, the dosage range was categorized into 4.6–8.3 mmol/d (285–515 mg/d of NO_3_^−^) and 8.3–16.4 mmol/d (515–1017 mg/d of NO_3_^−^). High-dose beetroot juice supplementation (8.3–16.4 mmol/d) produced a significant improvement in physical performance (*p* = 0.029), although the effect size was negligible (SMD = 0.14). In contrast, the low-dose group (4.6–8.3 mmol/d) did not achieve significant improvement (*p* = 0.061) and exhibited substantial heterogeneity (*I*^2^ = 65.6%). Taken together, high doses of supplementation are more recommended in this study. Although the high-dose effect size is marginal, even such minor differences can influence competitive outcomes in elite sports [2]. In addition, the safety of beetroot juice as a natural diet is guaranteed, and higher doses of supplementation are not harmful to the body. This conclusion aligns with the dose–response findings of Wylie et al. [8], and similarly, Hoon et al. [129] demonstrated that an 8.4 mmol dose improved 2000 m rowing performance, whereas a 4.2 mmol dose did not.

This study classified supplementation protocols into two modes: acute (administered 2–3 h prior to exercise) and chronic (administered more than 3 days prior to exercise). Although both approaches produced statistically significant improvement in physical performance, their effect sizes differed substantially: the acute supplementation group demonstrated a clear benefit (SMD = 0.20), whereas the chronic supplementation group exhibited a minimal effect (SMD = 0.13). Several considerations support the superiority of acute supplementation. First, its timing (2–3 h before exercise) aligns closely with the pharmacokinetics of dietary nitrate: bioavailability approaches 100%, and plasma nitrate concentrations peak approximately two hours post-ingestion (t_1_/_2_ ≈ 6 h) [9]. Notably, the International Olympic Committee (IOC) also recommends supplementing with 5–9 mmol of nitrate 2–3 h before exercise [2]. Second, the immediacy of acute dosing confers practical advantages in real-world settings. However, it is noteworthy that, despite its marginal effect size, chronic supplementation demonstrates the most robust statistical result (*I*^2^ = 27.1%), whereas acute supplementation exhibited positive heterogeneity (*I*^2^ = 67.1%). This suggests that, although acute dosing yields greater average gains, its effects are more variable, whereas chronic dosing provides greater stability. These findings need not be mutually exclusive: we hypothesize that layering an acute 2–3 h pre-competition dose onto a multiday chronic regimen may optimize performance benefits. Nonetheless, the ideal supplementation schedule for beetroot juice warrants further investigation.

Although this study suggests a nitrate level of 8.3–16.4 mmol (515–1017 mg/d NO_3_^−^) by supplementing with beetroot juice acute (2–3 h before exercise) or chronic supplementation (≥3 days) in order to improve physical performance, this represents only one effective strategy for reference; the optimal supplementation regimen warrants further investigation.

### 4.6. Study Limitations

Despite the comprehensive analysis conducted in this study on the effects of beetroot juice on physical performance in both athletes and healthy individuals, several limitations must be acknowledged. First, within the included meta-analyses, the duplication rate of all original data was 7.07%, and duplicate original studies were not excluded. This may have led to certain study results being over-weighted, potentially affecting the accuracy and reliability of the overall conclusions. Second, high heterogeneity was observed in specific outcome measures, such as the Yo-Yo IR1 test and time to exhaustion (TTE), which could be attributed to variations in experimental design, participant characteristics, beetroot juice dosage, or intervention duration across studies. Such high heterogeneity limits the generalizability of the findings and underscores the need for future research to further standardize experimental conditions to reduce variability. These limitations should be carefully considered when interpreting and applying the results of this study.

## 5. Conclusions

Acute (2–3 h pre-exercise) and chronic (≥3 days) supplementation strategies with beetroot juice to achieve nitrate levels of 8.3–16.4 mmol (515–1017 mg/d) are recommended to enhance physical performance. Beetroot juice shows population-specific effects: proper beetroot juice supplementation improves muscular strength in professional athletes and aerobic endurance in non-athletes. Moreover, appropriate supplementation of beetroot juice can improve the lactate tolerance in healthy adults.

## Figures and Tables

**Table 1:** Characteristics of included meta-analyses.

Name and Year	Include Studies	Population	Interventions	Dose/Time	Physical Performance Evaluation Methods	SMD (95% CI)	AMSTAR-2
(Van De Walle & Vukovich, 2018) [62]	29	Untrained individuals and trained individuals	Running, cycling, rowing, kayaking, team sports (soccer and rugby), and interval sprints, among others	N/A	MAIN: TT SUB: ATH, NON	TT, MAIN −0.05 (0.17 −0.28) ATH, SUB 0.1 (0.61 −0.41) NON, SUB 0.32 (0.47 0.1)	M
(Campos et al., 2018) [53]	53	Non-athletes (physically active) and athletes	Long-term open-ended test, long-duration test versus graded exercise test, and short-term test	N/A	SUB: ATH	ATH, SUB 0.04 (0.15 −0.05) ATH, SUB 0.25 (0.38 0.11)	M
(Evangelista et al., 2024) [58]	27	Healthy adult males, exercisers, and athletes	Isotonic: exercises such as squats and bench presses. Isokinetic, e.g., knee extension and flexion. Isometric Exercise intensity includes maximum repetitions (1RM) and repetitions to exhaustion	DOSE: Muscular Endurance ≤ 6.4 mmol/d, muscle endurance > 6.4 mmol/d TIME: 2–3 h before muscular endurance exercise, muscular endurance exercise after 3–8 days; 2–3 h before muscle strength exercise, muscle strength exercise after 3–8 days	MAIN: ME, MS SUB: ATH, NON, DOSE, TIME	MS, MAIN 0.26 (0.48 0.03) ME, MAIN 0.31 (0.51 0.1) ATH, SUB 0.48 (0.88 0.08) ATH, SUB 0.5 (0.85 0.14) NON, SUB 0.04 (0.68 −0.6) DOSE, SUB 0.57 (0.91 0.17) DOSE, SUB 0.17 (0.42 −0.08) TIME, SUB 0.24 (0.49 0) TIME, SUB 0.04 (0.79 0.01) TIME, SUB 0.2 (0.55 −0.16) TIME, SUB 0.33 (0.64 0.02)	H
(Coggan et al., 2021) [63]	19	Healthy college students, trained athletes	Exercises with large muscle groups (such as sprint riding) Exercises for small muscle groups (e.g., knee extensions)	TIME: acute muscle strength exercise, muscle strength exercise after 5–6 days	SUB: TIME	TIME, SUB 0.54 (0.71 0.37) TIME, SUB 0.25 (0.49 0.01)	M
(Alvares et al., 2022) [64]	34	Physically active people, athletes and non-training individual	Isometric contraction, isotonic contraction. The knee extensors were the most tested muscle group (18 studies), followed by the forearm flexors (11 studies)	TIME: 1.5–4 h before muscle strength exercise, muscle strength exercise after 4–8 days; 1.5–4 h before muscle endurance exercise, muscle endurance exercise after 4–8 days	MAIN: MS, ME SUB: ATH, NON, TIME	MS, MAIN 0.08 (0.15 0.01) ME, MAIN 0.31 (0.38 0.23) ATH, SUB 0.2 (0.42 −0.02) ATH, SUB 0.27 (0.82 −0.28) NON, SUB 0.08 (0.2 −0.05) NON, SUB 0.18 (1.13 −0.07) TIME, SUB 0.12 (0.24 −0.01) TIME, SUB 0.04 (0.13 −0.05) TIME, SUB 0.25 (0.45 0.05) TIME, SUB 0.42 (0.71 0.12)	M
(Hogwood et al., 2023) [65]	9	Healthy adult	Sprint interval training, high-intensity high-volume training, high-intensity interval training, and high-intensity endurance training	N/A	MAIN: TTE, MAIN SUB: VO2max	TTE, MAIN 0.08 (0.37 −0.21) VO2max, MAIN0.26 (0.57 −0.05)	H
(Esen et al., 2023) [51]	19	Elite athletic and healthy adults	Isometric exercises, isokinetic exercises, Wingate tests (e.g., 30 s full ride or running sprints), repetitive sprint tests (e.g., 4 × 20 s cycling sprints), and inertia load squats	Time: 2–3 h before peak power output exercise, 2–3 h before mean power output exercise, 2–3 h before time to peak power exercise	MAIN: MS SUB: TIME	MS, MAIN 0.25 (0.48 0.02) MS, MAIN 0.03 (0.74 −0.86) TIME, SUB 0.25 (0.48 0.02) TIME, SUB 0.28 (0.53 0.03) TIME, SUB −0.78 (−0.43 −1.14)	M
(Alsharif et al., 2023) [59]	25	Healthy adults, and athletes.	Cycling, running, canoeing, tennis, fighting, speed skating, cross-fit and sprinting	DOSE: <8 mmol/d average power output, ≥8 mmol/d average power output TIME: Single-day before total work exercise, total work exercise after multi-day (3–7 d); Single-day before average power output, average power output after Multi-day (3–7 d)	MAIN: MS, YY SUB: DOSE, TIME	MS, MAIN 0.01 (0.08 −0.06) YY, MAIN 0.17 (0.24 0.09) DOSE, SUB −0.14 (0.09 −0.37) DOSE, SUB 0.23 (0.49 −0.03) DOSE, SUB 0.19 (0.4 −0.02) DOSE, SUB 0.27 (0.54 0.01) TIME, SUB −0.1 (0.07 −0.28) TIME, SUB 0.34 (0.6 0.09) TIME, SUB 0.12 (0.26 −0.03) TIME, SUB 0.27 (0.54 0.01)	M
(Tan et al., 2024) [66]	6	Adult men and women healthy and actively involved in recreational sports.	30 s of all-out sprint test, repeat short sprint test	DOSE: 11.2–13 mmol, 5.6–8.3 mmol TIME: Mean Power Output after 5–6 d, 2.5–3 h before Mean Power Output; Peak Power Output exercise after 5–6 d, 2.5–3 h before peak power output	MAIN: MS SUB: DOSE, TIME	MS, MAIN 0.14 (0.349 −0.073) DOSE, SUB −0.17 (0.34 −0.38) DOSE, SUB 0.29 (0.59 0.01) DOSE, SUB 0.01 (0.35 −0.33) DOSE, SUB 0.2 (0.48 −0.05) TIME, SUB −0.02 (0.34 −0.38) TIME, SUB 0.29 (0.59 −0.01) TIME, SUB 0.05 (0.49 −0.39) TIME, SUB 0.16 (0.41 −0.08)	H
(Tan et al., 2023) [67]	6	Adult male	Squats with free weights, smith machine, flywheel machine mode; bench press is available in free weights and smith machine methods.	N/A	MAIN: MS	MS, MAIN 0.204 (0.411 −0.004)	M
(Wong et al., 2022) [68]	24	Active adults, recreational exercisers, and athletes	Time Trial (TT): Running, cycling, skiing, and rowing	TIME: 2–3 h before endurance exercise (exercise duration 30 min), endurance time exercise after 3–15 days (exercise duration 30 min); 2–3 h before endurance exercise (exercise duration 60 min), endurance time exercise after 3–15 days (exercise duration 60 min)	MAIN: TT SUB: TIME	TT, MAIN 0.15 (0.31 0) TT, MAIN 0.13 (0.47 −0.2) TIME, SUB 0.1 (0.28 −0.08) TIME, SUB 0.3 (0.59 0) TIME, SUB 0.03 (0.52 −0.46) TIME, SUB 0.22 (0.68 −0.23)	M
(Wong et al., 2021) [69]	17	Healthy adults, athletes, and athletic/trained individuals.	High-intensity interval training (HIIT), Treadmill sprinting, Kayak sprinting, and Yo-Yo IR1	TIME: 2–3 h before Average power output exercise, Average power output exercise after 3–6 d; 2–3 h before Peak Power Output, Peak Power Output exercise after 5–6 d	MAIN: MS, YY SUB: TIME	MS, MAIN 0.08 (0.3 −0.14) YY, MAIN 0.46 (0.77 0.14) TIME, SUB −0.18 (0.31 −0.66) TIME, SUB 0.04 (0.4 −0.31) TIME, SUB 0.05 (0.37 −0.26) TIME, SUB 0.11 (0.41 −0.2)	M
(McMahon et al., 2017) [70]	47	Healthy human adolescents or adults.	Endurance Exercise Tests: Includes time trial (TT), Exhaustion Time Test (TTE), and Increasing Load Exercise Test (GXT).	N/A	MAIN: TT, TTE	TT, MAIN −0.1 (0.06 −0.27) TTE, MAIN 0.33 (0.5 0.15) VO2max, MAIN 0.25 (0.56 −0.06)	H
(Hoon et al., 2013) [71]	17	Active adults.	Exhaustion test (GXT), constant load exercise to exhaustion test (TTE), endurance exercise time/distance (i.e., time trial [TT]), and one study used a repeat time trial design (6×500m time trial). Exercise programs include cycling, running, rowing, leg stretching, arm cranking, etc.	N/A	MAIN: TT, TTE, VO2max	TT, MAIN 0.11 (0.37 −0.16) TTE, MAIN 0.79 (1.35 0.23) VO2max, MAIN 0.26 (0.62 −0.1)	M
(Gao et al., 2021) [72]	73	Healthy adults, and elite athletes.	Various endurance exercise forms, such as running, cycling, swimming, rowing, etc., the types of exercise tests include constant load exercise to exhaustion, increasing load exercise to exhaustion, time trial, etc.	N/A	MAIN: TT, TTE SUB: ATH, NON	TT, MAIN −0.08 (0.04 −0.21) TTE, MAIN 0.47 (0.64 0.3) ATH, SUB 0.05 (0.3 −0.2) NON, SUB 0.09 (0.56 −0.38)	M

Abbreviations: SMD, Standardized Mean Difference; N/A, Not Available; AMSTAR-2, A Measurement Tool To Assess Systematic Reviews; H, High Rating Overall Confidence; M, Moderate Rating Overall Confidence; YY, Yo-Yo Intermittent Recovery Level 1 Test; TT, Time Trial Performance; TTE, Time To Exhaustion In Seconds; MS, Muscle Strength; ME, Muscle Endurance; VO2max, Maximal Oxygen Uptake; ATH, Athlete; NON, Non-Athletes; DOSE, Dose Of Intervention; TIME, Time Of Intervention; h, Hour; d, Day; MAIN, Main Study Group; SUB, Sub Study Group.

**Figure A1:**
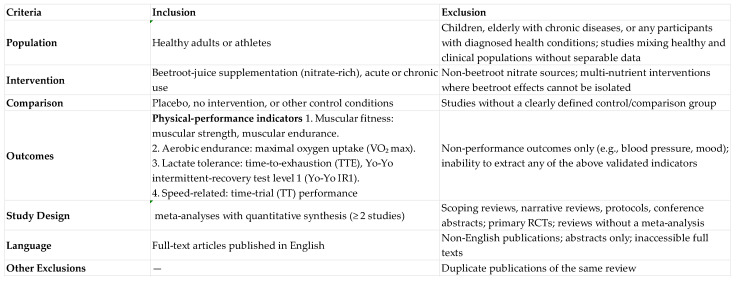
Inclusion and Exclusion Criteria.

## Data Availability

The data presented in this study are available on request from the corresponding author. The data are not publicly available due to privacy.

## Supplementary Materials

The following supporting information can be downloaded at: https://www.mdpi.com/article/10.3390/nu17121958/s1, File S1: Search Strategy; File S2: AMSTAR-2 Table; File S3: Forest and Funnel Plot; File S4: Corrected Covered Area; File S5: Data Synthesis; File S6: PRISMA_2020_checklist; File S7: PRISMA_2020_abstract_checklist. Reference [130] is cited in Supplementary Materials.
