# Supplementary material for: Effects of Beetroot Juice on Physical Performance in Professional Athletes and Healthy Individuals: An Umbrella Review
Source: Nutrients. 2025 Jun 9;17(12):1958. doi: 10.3390/nu17121958 (PMC12195723; doi:10.3390/nu17121958)
Supplement: Supplementary file 1 [file nutrients-17-01958-s001.zip › Supplementary File S1 Search Strategy.pdf]

Supplementary Table Search Strategy

PubMed

Search: ((((("Exercise"[Mesh] OR "Motion"[Mesh]) OR (Exercises OR Exercise, Physical OR Physical Exercise OR Physical Activity OR Activities, Physical OR Exercise, Aerobic OR Aerobic Exercises OR Exercises, Aerobic OR Exercise, Isometric OR Exercises, Isometric OR Isometric Exercise OR Exercises, Acute OR Exercise Training OR Training, Exercise)) OR ("Athletes"[Mesh] OR Athlete OR Professional Athletes OR Athlete, Professional OR Athletes, Professional OR Professional Athlete OR Elite Athletes OR Athlete, Elite OR Athletes, Elite OR Elite Athlete OR College Athletes OR Athlete, College OR Athletes, College OR College Athlete)) AND ("Beta vulgaris"[Mesh] OR Beta vulgari OR vulgari, Beta OR beet OR beetroot OR beet juice OR beetroot juice OR beets OR beetroots OR beets juice OR beetroots juice OR beets juices OR beetroots juices OR Beta vulgaris powder OR beet powder OR beetroot powder OR Beta vulgaris supplements OR beet supplements OR beetroot supplements OR Beta vulgaris supplement OR beet supplement OR beetroot supplement OR red beet)) OR ("Nitrates"[Mesh] OR nitrate OR nitrates OR NO3-)) AND ("Meta-Analysis" [Publication Type] OR meta-analysis[Title/Abstract] OR "Systematic Review" [Publication Type] OR Systematic Review[Title/Abstract])

Web of science：

TS=(Motion OR Exercises OR Exercise, Physical OR Physical Exercise OR Physical Activity OR Activities, Physical OR Exercise, Aerobic OR Aerobic Exercises OR Exercises, Aerobic OR Exercise, Isometric OR Exercises, Isometric OR Isometric Exercise OR Exercises, Acute OR Exercise Training OR Training, Exercise OR Athlete OR Professional Athletes OR Athlete, Professional OR Athletes, Professional OR Professional Athlete OR Elite Athletes OR Athlete, Elite OR Athletes, Elite OR Elite Athlete OR College Athletes OR Athlete, College OR Athletes, College OR College Athlete) and Preprint Citation Index

AND

TS=(Beta vulgaris OR Beta vulgaris OR vulgaris, Beta OR beet OR beetroot OR beet juice OR beetroot juice OR beets OR beetroot OR beets juice OR beetroot juice OR beets juices OR beetroot juices OR Beta vulgaris powder OR beet powder OR beetroot powder OR Beta vulgaris supplements OR beet supplements OR beetroot supplements OR Beta vulgaris supplement OR beet supplement OR beetroot supplement OR red beet OR nitrates OR nitrate OR NO3) and Preprint Citation Index

AND

TS=(Meta-Analysis OR Systematic Review) and Preprint Citation Index

Cochrane

MeSH descriptor: [Athletes] explode all trees OR

MeSH descriptor: [Exercise] explode all trees OR

OR

(Athlete):ti,ab,kw OR (Professional Athletes):ti,ab,kw OR (Athlete, Professional):ti,ab,kw OR (Athletes, Professional):ti,ab,kw OR (Professional Athlete):ti,ab,kw OR (Elite Athletes):ti,ab,kw OR (Athlete, Elite):ti,ab,kw OR (Athletes, Elite):ti,ab,kw OR (Elite Athlete):ti,ab,kw OR (College Athletes):ti,ab,kw OR (Athlete, College):ti,ab,kw OR (Athletes, College):ti,ab,kw OR (College Athlete):ti,ab,kw

OR

MeSH descriptor: [Motion] explode all trees

OR

(Motion):ti,ab,kw OR (Exercises):ti,ab,kw OR (Exercise, Physical):ti,ab,kw OR (Physical Exercise):ti,ab,kw OR (Physical Activity):ti,ab,kw OR (Activities, Physical):ti,ab,kw OR (Exercise, Aerobic):ti,ab,kw OR (Aerobic Exercises):ti,ab,kw OR (Exercises, Aerobic):ti,ab,kw OR (Exercise, Isometric):ti,ab,kw OR (Exercises, Isometric):ti,ab,kw OR (Isometric Exercise):ti,ab,kw OR (Exercises, Acute):ti,ab,kw OR (Exercise Training):ti,ab,kw OR (Training, Exercise):ti,ab,kw

AND

MeSH descriptor: [Beta vulgaris] explode all trees

OR

(Beta vulgari):ti,ab,kw OR (vulgari, Beta):ti,ab,kw OR (Chard):ti,ab,kw OR (Chards):ti,ab,kw OR (beet):ti,ab,kw OR (beetroot):ti,ab,kw OR (beet juice):ti,ab,kw OR (beetroot juice):ti,ab,kw OR (beets):ti,ab,kw OR (beetroots):ti,ab,kw OR (beets juice):ti,ab,kw OR (beetroots juice):ti,ab,kw OR (beets juices):ti,ab,kw OR (beetroots juices):ti,ab,kw OR (Beta vulgaris powder):ti,ab,kw OR (beet powder):ti,ab,kw OR (beetroot powder):ti,ab,kw OR (Beta vulgaris supplements):ti,ab,kw OR (beet supplements):ti,ab,kw OR (beetroot supplements):ti,ab,kw OR (Beta vulgaris supplement):ti,ab,kw OR (beet supplement):ti,ab,kw OR (beetroot supplement):ti,ab,kw OR (red beet):ti,ab,kw OR (red beets):ti,ab,kw OR (red beetroot):ti,ab,kw

OR

MeSH descriptor: [Nitrates] explode all trees

OR

(Nitrates):ti,ab,kw OR (nitrates):ti,ab,kw

Sportdiscus and CINAHL

Motion OR Exercises OR Exercise, Physical OR Physical Exercise OR Physical Activity OR Activities, Physical OR Exercise, Aerobic OR Aerobic Exercises OR Exercises, Aerobic OR Exercise, Isometric OR Exercises, Isometric OR Isometric Exercise OR Exercises, Acute OR Exercise Training OR Training, Exercise OR Athlete OR Professional Athletes OR Athlete, Professional OR Athletes, Professional OR Professional Athlete OR Elite Athletes OR Athlete, Elite OR Athletes, Elite OR Elite Athlete OR College Athletes OR Athlete, College OR Athletes, College OR College Athlete

AND

Beta vulgaris OR Beta vulgari OR vulgari, Beta OR beet OR beetroot OR beet juice OR beetroot juice OR beets OR beetroots OR beets juice OR beetroots juice OR beets juices OR beetroots juices OR Beta vulgaris powder OR beet powder OR beetroot powder OR Beta vulgaris supplements OR beet supplements OR beetroot supplements OR Beta vulgaris supplement OR beet supplement OR beetroot supplement OR red beet OR nitrates OR nitrate OR NO3

AND

Meta-Analysis OR Systematic Review

scopus

((TITLE-ABS-KEY (motion) OR TITLE-ABS-KEY (exercises) OR TITLE-ABS-KEY (physical) OR TITLE-ABS-KEY (athlete) OR TITLE-ABS-KEY (elite) OR TITLE-ABS-KEY (aerobic) OR TITLE-ABS-KEY (isometric) OR TITLE-ABS-KEY (acute) OR TITLE-ABS-KEY (professional AND athlete) OR TITLE-ABS-KEY (elite AND athletes) OR TITLE-ABS-KEY (training) OR TITLE-ABS-KEY (professional))) AND ((TITLE-ABS-KEY (beta AND vulgaris) OR TITLE-ABS-KEY (beet) OR TITLE-ABS-KEY (beetroot) OR TITLE-ABS-KEY (beetroot AND juice) OR TITLE-ABS-KEY (beetroot AND extract) OR TITLE-ABS-KEY (beet AND powder) OR TITLE-ABS-KEY (beetroot AND powder) OR TITLE-ABS-KEY (beet AND supplements) OR TITLE-ABS-KEY (beetroot AND supplements) OR TITLE-ABS-KEY (nitrates) OR TITLE-ABS-KEY (nitrate) OR TITLE-ABS-KEY (no3) OR TITLE-ABS-KEY (beetroot AND supplement) OR TITLE-ABS-KEY (beet AND supplement))) AND ((TITLE-ABS-KEY (meta-analysis) OR TITLE-ABS-KEY (systematic AND review) OR TITLE-ABS-KEY (meta AND analysis) OR TITLE-ABS-KEY (systematic AND reviews))) AND PUBYEAR > 1999 AND PUBYEAR < 2025

Embase

motion:ti,ab,kw OR exercises:ti,ab,kw OR (exercise,:ti,ab,kw AND physical:ti,ab,kw) OR (physical:ti,ab,kw AND exercise:ti,ab,kw) OR (physical:ti,ab,kw AND activity:ti,ab,kw) OR (activities,:ti,ab,kw AND physical:ti,ab,kw) OR (exercise,:ti,ab,kw AND aerobic:ti,ab,kw) OR (aerobic:ti,ab,kw AND exercises:ti,ab,kw) OR (exercises,:ti,ab,kw AND aerobic:ti,ab,kw) OR (exercise,:ti,ab,kw AND isometric:ti,ab,kw) OR (exercises,:ti,ab,kw AND isometric:ti,ab,kw) OR (isometric:ti,ab,kw AND exercise:ti,ab,kw) OR (exercises,:ti,ab,kw AND acute:ti,ab,kw) OR (exercise:ti,ab,kw AND training:ti,ab,kw) OR (training,:ti,ab,kw AND exercise:ti,ab,kw) OR athlete:ti,ab,kw OR (professional:ti,ab,kw AND athletes:ti,ab,kw) OR (athlete,:ti,ab,kw AND professional:ti,ab,kw) OR (athletes,:ti,ab,kw AND professional:ti,ab,kw) OR (professional:ti,ab,kw AND athlete:ti,ab,kw) OR (elite:ti,ab,kw AND athletes:ti,ab,kw) OR (athlete,:ti,ab,kw AND elite:ti,ab,kw) OR (athletes,:ti,ab,kw AND elite:ti,ab,kw) OR (elite:ti,ab,kw AND athlete:ti,ab,kw) OR (college:ti,ab,kw AND athletes:ti,ab,kw) OR (athlete,:ti,ab,kw AND college:ti,ab,kw) OR (athletes,:ti,ab,kw AND college:ti,ab,kw) OR (college:ti,ab,kw AND athlete:ti,ab,kw)

AND

beta:ti,ab,kw OR vulgari:ti,ab,kw OR (vulgari,:ti,ab,kw AND beta:ti,ab,kw) OR chard:ti,ab,kw OR chards:ti,ab,kw OR beet:ti,ab,kw OR beetroot:ti,ab,kw OR (beet:ti,ab,kw AND juice:ti,ab,kw) OR (beetroot:ti,ab,kw AND juice:ti,ab,kw) OR beets:ti,ab,kw OR beetroots:ti,ab,kw OR (beets:ti,ab,kw AND juice:ti,ab,kw) OR (beetroots:ti,ab,kw AND juice:ti,ab,kw) OR (beets:ti,ab,kw AND juices:ti,ab,kw) OR (beetroots:ti,ab,kw AND juices:ti,ab,kw) OR (beta:ti,ab,kw AND vulgaris:ti,ab,kw AND powder:ti,ab,kw) OR (beet:ti,ab,kw AND powder:ti,ab,kw) OR (beetroot:ti,ab,kw AND powder:ti,ab,kw) OR (beta:ti,ab,kw AND vulgaris:ti,ab,kw AND supplements:ti,ab,kw) OR (beet:ti,ab,kw AND supplements:ti,ab,kw) OR (beetroot:ti,ab,kw AND supplements:ti,ab,kw) OR (beta:ti,ab,kw AND vulgaris:ti,ab,kw AND supplement:ti,ab,kw) OR (beet:ti,ab,kw AND supplement:ti,ab,kw) OR (beetroot:ti,ab,kw AND supplement:ti,ab,kw) OR (red:ti,ab,kw AND beet:ti,ab,kw) OR (red:ti,ab,kw AND beets:ti,ab,kw) OR (red:ti,ab,kw AND beetroot:ti,ab,kw) OR nitrates:ti,ab,kw OR nitrate:ti,ab,kw OR no3:ti,ab,kw

AND

'meta-analysis protocol':ti,ab,kw OR 'meta analysis (topic)':ti,ab,kw OR 'meta analysis':ti,ab,kw OR 'systematic review':ti,ab,kw OR review:ti,ab,kw
