# Supplementary material for: Effects of Beetroot Juice on Physical Performance in Professional Athletes and Healthy Individuals: An Umbrella Review
Source: Nutrients. 2025 Jun 9;17(12):1958. doi: 10.3390/nu17121958 (PMC12195723; doi:10.3390/nu17121958)
Supplement: Supplementary file 1 [file nutrients-17-01958-s001.zip › Supplementary File S3 Forest and Funnel Plot.pdf]

Forest and Funnel plots of the effect of NO3- supplementation on each physical performance outcome

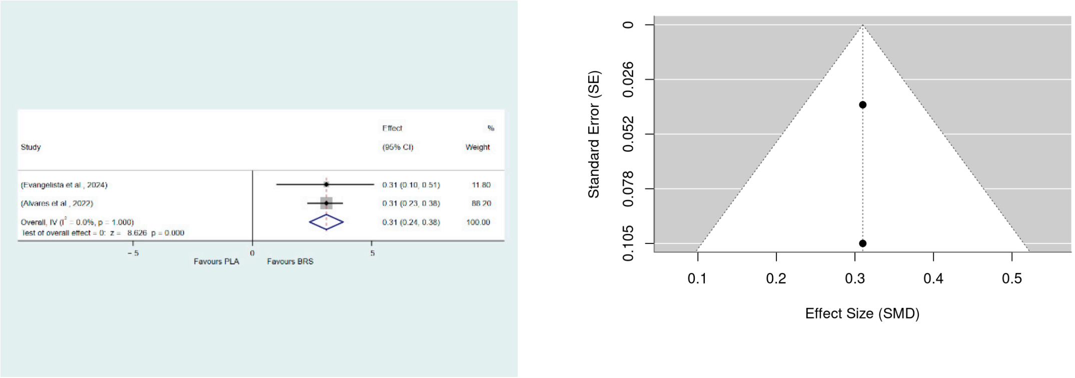

Muscle Endurance

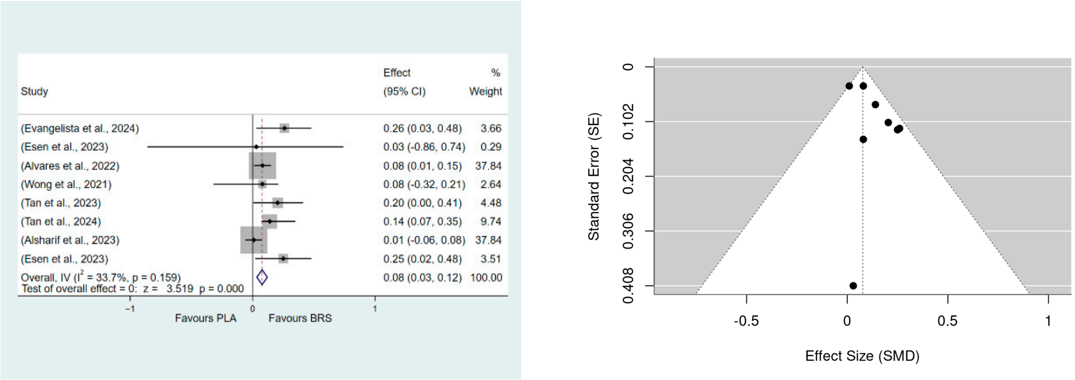

Muscle Strength

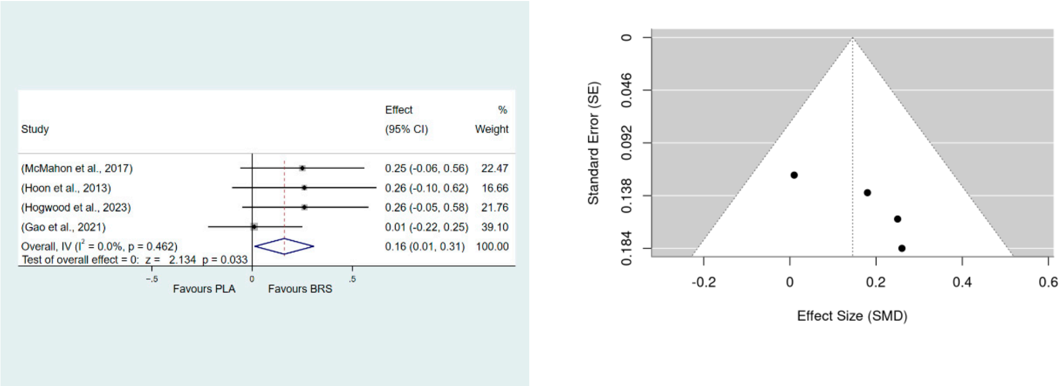

Maximal Oxygen Uptake (VO2max)

Forest and Funnel plots of the effect of NO3- supplementation on each physical performance outcome

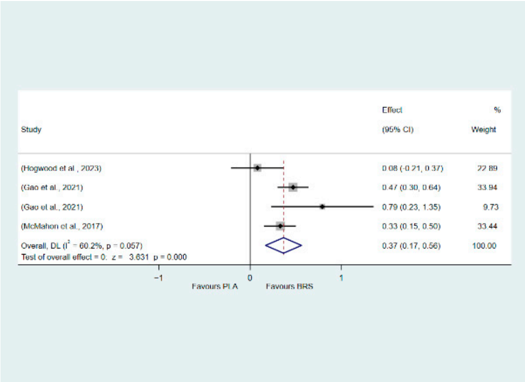

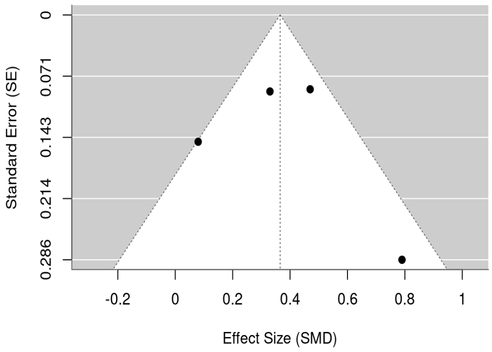

Time To Exhaustion in Seconds

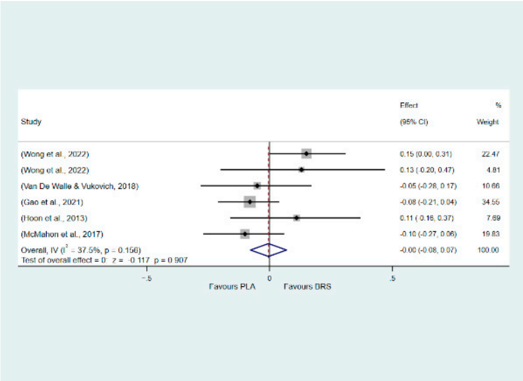

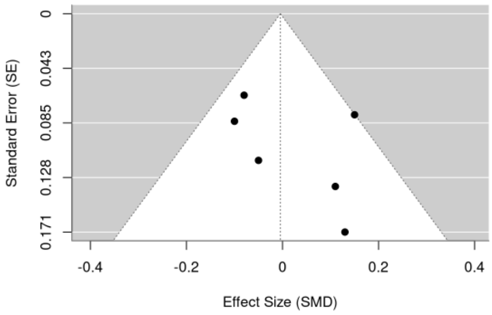

Time Trial Performance

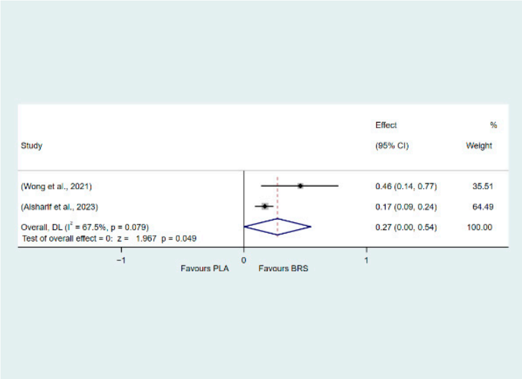

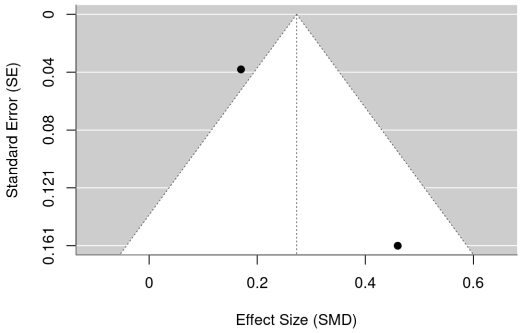

Yo-Yo IR1

Forest and Funnel plots of the effect of NO3- supplementation on each physical performance outcome

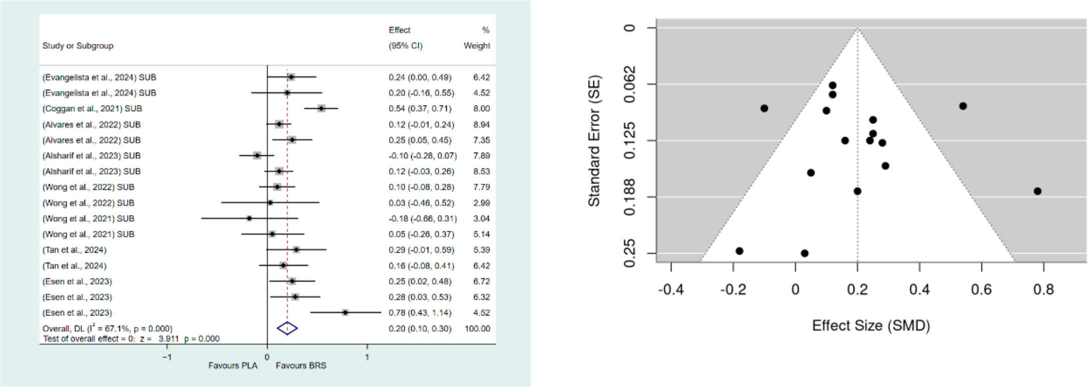

Acute Supplementation Physical Performance

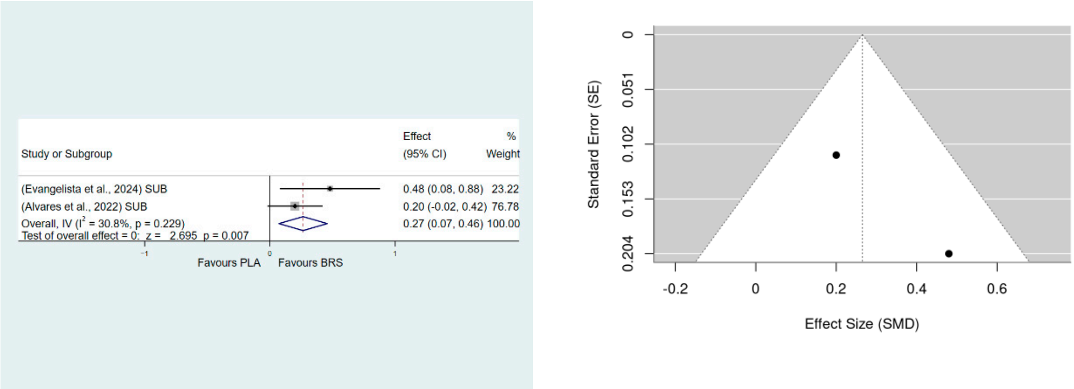

Professional Athletes Muscle Strength

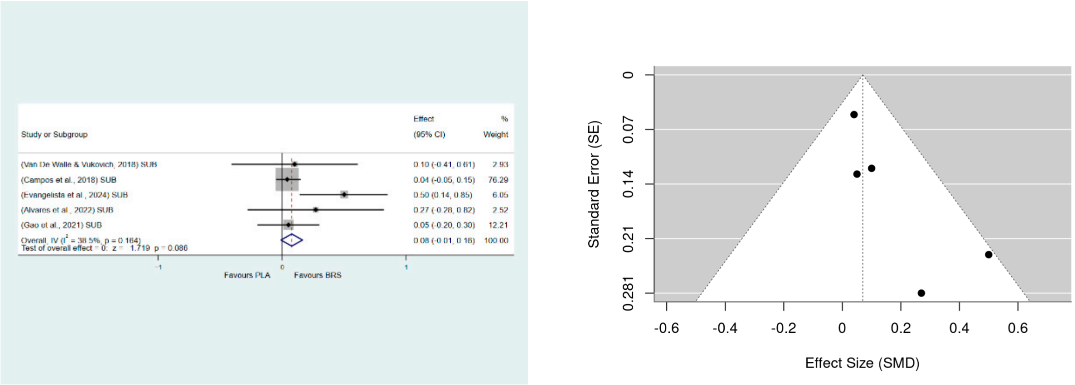

Professional Athletes Aerobic Endurance

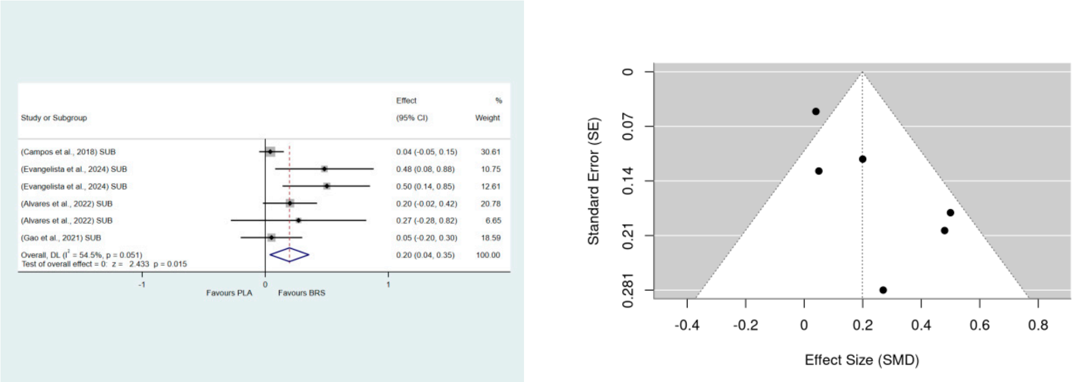

Professional Athletes Physical Performance

Forest and Funnel plots of the effect of NO3- supplementation on each physical performance outcome

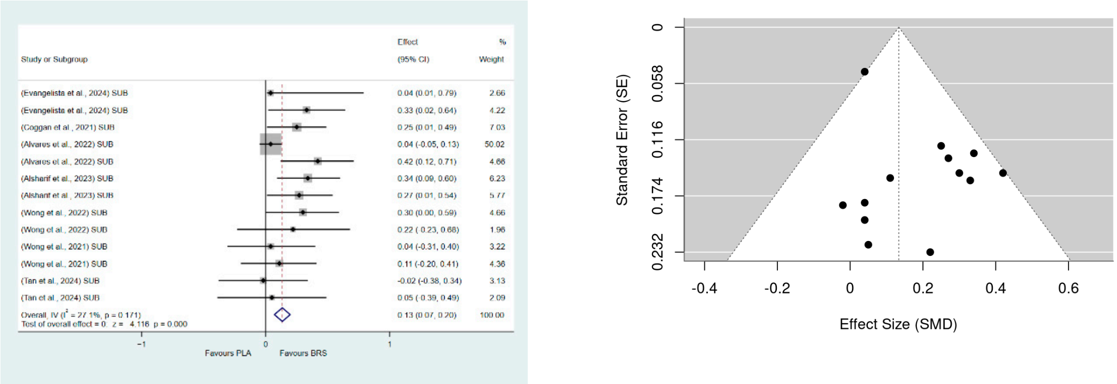

Chronic Supplementation Physical Performance

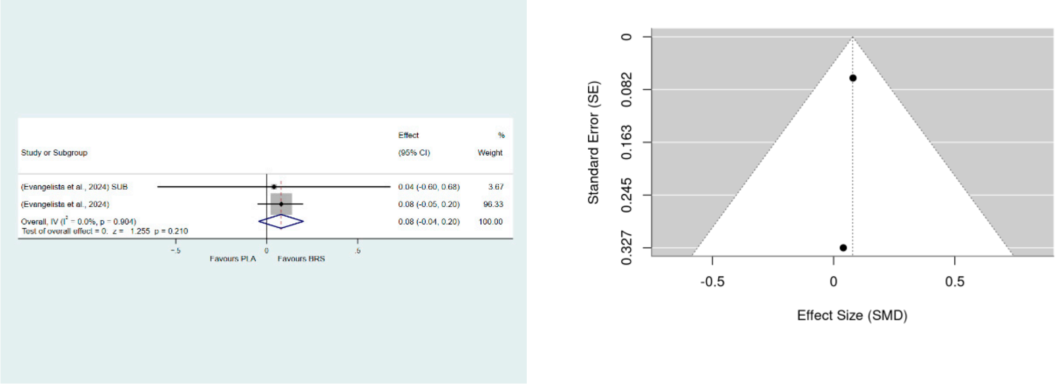

Non-athletes Muscle Strength

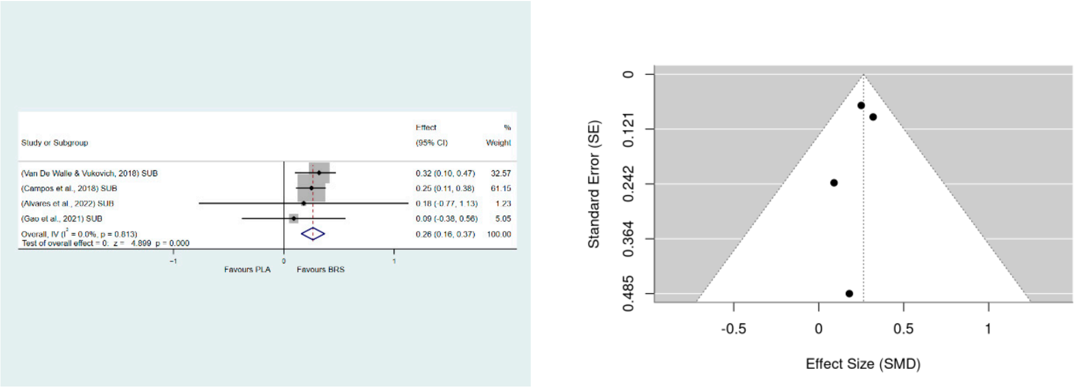

Non-athletes Aerobic Endurance

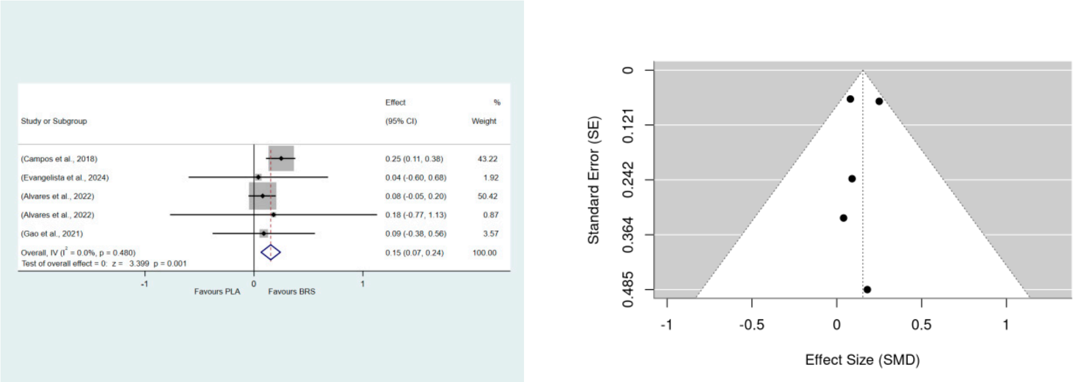

Non-athletes Physical Performance

Forest and Funnel plots of the effect of NO3- supplementation on each physical performance outcome

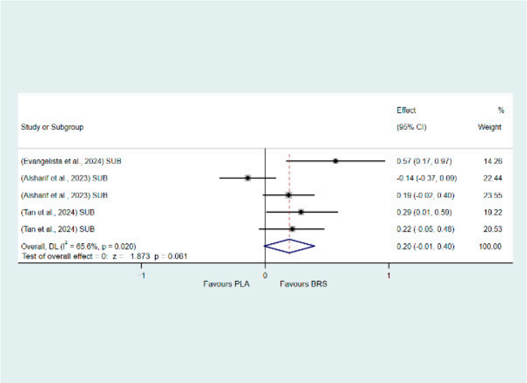

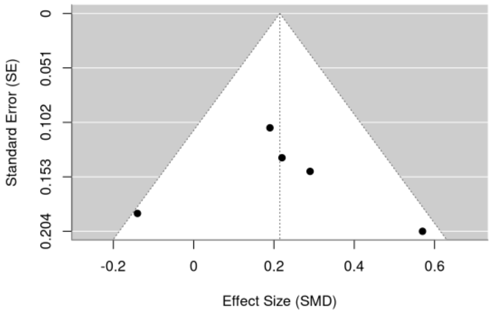

Dosage of 4.6–8.3 mmol/day

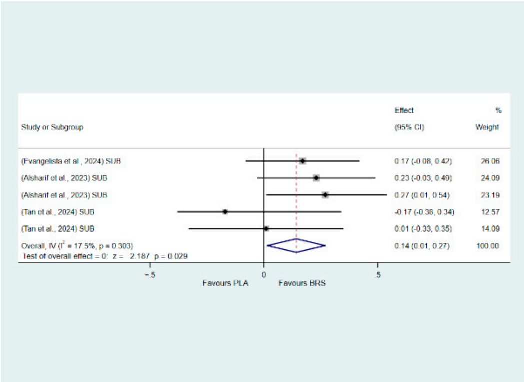

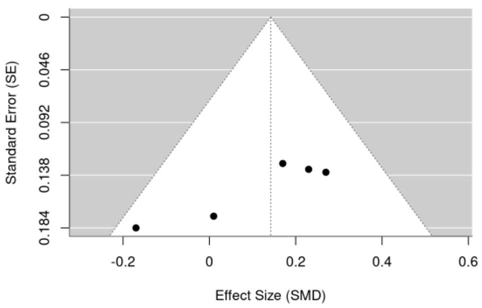

Dosage of 8.3–16.4 mmol/day
